# Epidemiological and molecular surveillance of norovirus in the Brazilian Amazon: description of recombinant genotypes and improvement of evolutionary analysis

**DOI:** 10.1590/S1678-9946202466022

**Published:** 2024-04-19

**Authors:** Jonaia Novaes da Costa, Jones Anderson Monteiro Siqueira, Dielle Monteiro Teixeira, Patrícia dos Santos Lobo, Sylvia de Fátima dos Santos Guerra, Isadora Monteiro Souza, Bruna Trindade Moreira Cardoso, Luana Silva Soares Farias, Hugo Reis Resque, Yvone Benchimol Gabbay, Luciana Damascena da Silva

**Affiliations:** 1 Instituto Evandro Chagas, Seção de Virologia, Ananindeua, Pará, Brazil

**Keywords:** Norovirus, Diarrhea, Gastroenteritis, Amazon Region, Molecular evolution

## Abstract

Noroviruses are highly infectious, genetically diverse viruses. Global outbreaks occur frequently, making molecular surveillance important for infection monitoring. This cross-sectional descriptive study aimed to monitor cases of norovirus gastroenteritis in the Brazilian Amazon. Fecal samples were tested by immunoenzymatic assay, RT-PCR and genetic sequencing for the ORF1/ORF2 and protease regions. Bayesian inference with a molecular clock was employed to construct the phylogeny. The norovirus prevalence was 25.8%, with a higher positivity rate among children aged 0-24 months. Genogroup GII accounted for 98.1% of the sequenced samples, while GI accounted for 1.9% of them. The GII.P16/GII.4 genotype was the most prevalent, with an evolution rate of 2.87x10^−3^ and TMRCA estimated in 2012. This study demonstrates that norovirus is a primary causative agent of gastroenteritis and provides data on viral genetic diversity that may facilitate infection surveillance and vaccine development.

## INTRODUCTION

Enteric viruses can be caused by a variety of pathogens, but norovirus (NoV) is recognized as one of the leading causes of gastroenteritis worldwide^1,2^. NoV is transmitted by direct contact with infected individuals, consumption of contaminated water and food, and fomites. It is estimated that more than 600 million people worldwide suffer from gastroenteritis due to NoV infection, resulting in an estimated 19,496 deaths annually (95% CI: 8,747–38,421)^3,4^. A significant number of these cases are in children aged under five years (10,629; 95% CI 5,274–19,582) or in older adults (aged over 70 years: 3,693; 95% CI 810–8,647), according to the Global Burden of Disease Study 2016^4^.

NoV, family *Caliciviridae*, are non-enveloped viruses with a single-stranded RNA genome organized into three open reading frames (ORF). ORF1 encodes the non-structural proteins p48, NTPase, p22, VPg, Pro and RdRp, while ORF2 and ORF3 encode the structural proteins VP1 and VP2, respectively^5^. These viruses are presented with a high genetic diversity and are classified into 10 genogroups (GI-GX) and 48 genotypes based on the sequences of the VP1 protein and RNA polymerase (RdRp)^6^.

Pandemic NoV strains have been identified since the 1990s^6^. Viral evolution has been linked to various mechanisms, including point mutations and genetic recombination, which often occurs in the overlap region of ORF1 and ORF2^5,7^. Viral genetic diversity contributes to the global spread of the virus and is a significant barrier to vaccine development^5^.

NoV circulates in different regions of the world, causing outbreaks and sporadic cases^8-10^. Meta-analyses showed that the global prevalence of NoV infection among children with gastroenteritis was 17.7%^11^. Over the years, there have been several advances in scientific research, including the diagnosis of NoV and the molecular characterization of circulating genotypes^12,13^.

Efforts to diagnose NoV based on the epidemiological surveillance of viral gastroenteritis in the Amazon began in 2010. Since then, relevant scientific progress have been made, including the etiological description and identification of circulating genetic variations^10,13^. Therefore, epidemiological surveillance of noroviruses is essential to minimize the impact of infections. This work aimed to determine the temporal trends of prevalence, genetic diversity and viral evolution of noroviruses, which are strategic considerations for the surveillance of viral diarrheal diseases in the Brazilian Amazon.

## MATERIALS AND METHODS

### Ethics approval statement

This study was approved by the Human Research Ethics Committee (protocol Nº 1.318.103).

### Study type and sample description

This study is a cross-sectional, retrospective and descriptive analysis of NoV infections in the Amazon. Gastroenteritis cases were identified based on the presence of diarrhea (three or more liquid or pasty bowel movements) accompanied by fever, vomiting and abdominal pain.

Clinical and epidemiological data of participants were collected from the Laboratory Environment Manager system. Stool samples from patients aged 0 to 70 years were collected in sentinel units of the Viral Gastroenteritis Surveillance Network (Ministry of Health) from 2018 to 2022.

Month and year of sample collection and patient age were analyzed. Continuous variables were analyzed for mean and standard deviation, whereas categorical variations were analyzed for absolute and relative frequencies. Sample size was determined by considering the sample population (number of samples received during the time frame), the expected infection frequency (based on prior research) and a 5% margin of error. Initial technical term abbreviations were explained for clarity. The data were organized into tables and graphs using Epi Info™ (version 7.2.5, CDC, USA) and Excel (2019, Microsoft Corporation, USA).

### Sample preparation and nucleic acid extraction

Fecal suspensions were prepared at 10% using 0.01M Tris HCl/Ca buffer++ (pH 7.2). They were then centrifuged at 4,500xg for 10 min and the supernatant was stored at –20 °C. Nucleic acids were extracted (140 µL fecal suspension) using the commercial QIAmp viral RNA kit (QIAgen, Hilden, Germany) according to the manufacturer’s instructions.

### Norovirus detection

For NoV diagnosis, fecal suspensions were analyzed by enzyme immunoassay (EIA) using the commercial third-generation Ridascreen^®^ Norovirus EIA kit (R-Biopharm, Germany), which is based on the capture of NoV antigens (genogroups I and II) by monoclonal antibodies, in accordance with the manufacturer’s instructions.

### Semi-nested PCR of the ORF1/ORF2 junction region

The samples positive by EIA were subjected to amplification of the junction region (ORF1/ORF2), which allows genotypic characterization, including recombinant strains. In the first step of the reaction, primers Mon 431/432 and G2SKR (amplicon: 544 bp) were used^14,15^. In the second step, a semi-nested reaction was performed with primers COG2F and G2SKR (amplicon: 390 bp), specific for the viral capsid^15,16^. These assays were performed using the SuperScript^®^ III OneStep RT-PCR System with the Platinum^®^ Taq DNA Polymerase Kit (Invitrogen^®^) according to the manufacturer’s recommendations.

### Primer design and validation for genome sequencing of the NoV protease region

Samples genotyped as GII.4 by the junction region were subjected to PCR for the protease region (ORF1). Specific primers for this PCR were designed based on multiple alignments in a database of NoV nucleotide sequences (genotypes GII.P31/GII.4 and GIIP16/GII.4) obtained from the National Center for Biotechnology Information (NCBI) database.

Primers were first designed using the Primer3 tool, available in the Geneious software (version R10, Dotmatics, New Zealand), considering the following parameters: 500-700 bp minimum product size of, 57-60 °C melting temperature and 55% mean GC content. Two methods were used to validate the RT-PCR (protease region):

- In silico approach: these primers were validated using the Oligo Analyzer tool, which describes physical properties such as complementary sequence, length, GC content, melting temperature, extinction coefficient and molecular weight. In addition, the prediction of dimer formation and secondary structures (hairpin) and specificity parameters (using the BLAST tool) were also analyzed. The primers were named Noro3064F (5’-TGGTTCAGGTTCAGGTTGGCTTTT-3’) and Noro3828R (5’-GCAGCTTCCAACACACTTGG-3).
- Laboratory conformity analysis (sensitivity and specificity): validation tests were performed using samples and controls (positive controls: GII.4 and non-GII.4 standard strains; negative controls: H_2_O-free DNAse/RNAse). Positive (N=6) and negative controls (N = 6) were tested in repeated runs in the same thermocycler (Veriti™ 96-well thermal cycler (Applied Biosystems, Massachusetts, United States) by two analysts, using a temperature gradient of 57-60 °C to determine the optimal annealing temperature. The Qubit™ Fluorometer (Invitrogen) was used for fluorometric quantification with the Qubit^®^ RNA BR Assay Kit (Invitrogen). Method performance was confirmed using the conformity hypothesis test, considering amplification in GII.4 and non-GII.4 standard strains.

RT-PCR reactions with a 25 μL final volume, consisting of 8 μL viral RNA and 17 μL reagent mix (1.5 μL H_2_O-free DNAse/RNAse, 12.5 μL 2X Reaction Mix, 1 μL of each primer 20µM, 1 μL SuperScript^®^ III RT/Platinum™ Taq Mix), were subjected to the following amplification conditions: 45 °C for 30 min; 94 °C for 2 min; followed by 40 cycles of 94 °C for 15 s, 60°C for 30 s and 68°C for 90 s, and lastly 68 °C for 5 min. Visualization of amplicons (703 bp) was performed on 1.5% agarose gels.

### Partial sequencing

The PCR-amplified samples were purified using the Wizard^®^ SV Gel and PCR Clean-Up System kit (Promega^®^) according to the manufacturer’s instructions, followed by nucleotide sequencing using the BigDye^®^ Terminator v 3.1 Cycle Sequencing kit (Applied Biosystems, Foster, USA) on the 3130XL DNA Sequencer platform (Applied Biosystems, Foster, USA).

### Genotyping and phylogenetic analysis

The sequences obtained after sequencing were genotyped using the Noro Genotyping Tool and the Basic Local Alignment Search Tool (BLAST). From these sequences, maximum likelihood phylogenetic analyses (based on nearest neighbor interactions and the minimum evolution criterion) and Bayesian phylogenetic inference were performed using FastTree and MrBayes, available in the Geneious program.

### Molecular evolution of the Pro region in NoV GII.P16/GII.4 strains

The time-scaled phylogenetic tree was constructed based on a dataset of complete VP1 sequences (n = 234) using the Bayesian Markov Chain Monte Carlo (MCMC) analysis in BEAST software (version v1.10.4, BEAST Developers, Europe and USA). The molecular clock (strict or relaxed) and coalescence model were chosen based on the PS/SS sampling method, using the Bayes factor^17^.

### Recombination analysis

Sequences from a specific region in ORF1/ORF2 with divergent genotypes in ORF1 and ORF2 were analyzed using the Recombination Detection Program (RDP4, version 4.101, University of Manchester, United Kingdom) to confirm recombination events^18^.

### Statistical analysis

Nonparametric analyses were performed using the chi-squared test and the G-test; odds ratio tests were also performed to calculate the advantage or disadvantage of the results for NoV in relation to age groups. These tests were performed in Bioestat (version 5.3, Instituto Mamiraua, Brazil) and Jamovi (version 2.3, The Jamovi Project, Australia) softwares. For temporal distribution, inflection point regression analysis was performed using Joinpoint Regression to assess the trend and calculate APC/MPC (annual/monthly percentage changes). Data were analyzed considering a 5% significance level and 95% confidence interval (CI).

## RESULTS

From January 2018 to December 2022, 695 fecal samples from the Viral Gastroenteritis Surveillance Network were processed. Of these, 25.8% (179/695) were positive for NoV. The prevalence of NoV was 28.5% (55/193) in 2018, 18.3% (26/142) in 2019, 10.6% (5/47) in 2020, 30.3% (64/211) in 2021 and 28.4% (29/102) in 2022.

**Figure 1:**
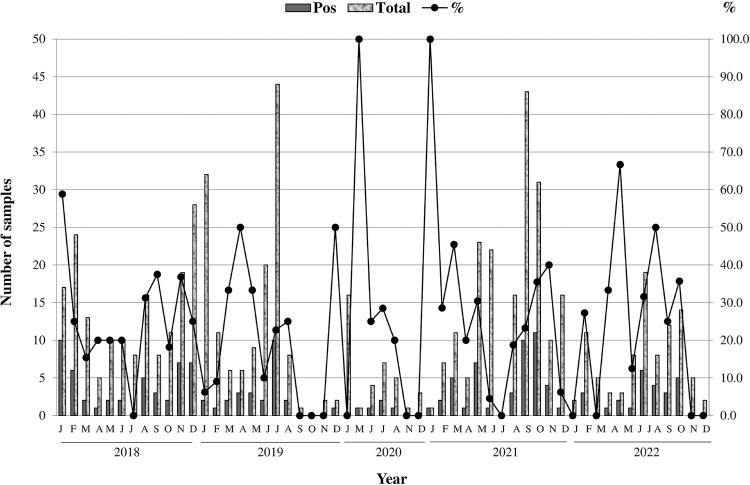
Temporal distribution of NoV prevalence in gastroenteritis cases in samples from the Viral Gastroenteritis Surveillance Network during the period from January 2018 to December 2022.

As for the temporal distribution (considering the information available when the samples were collected), it was observed that the months with the highest positivity were January 2018 (58.8% - 10/17), March 2021 (45.4% - 5/11), November 2021 (40% - 4/10), October 2021 (35.5% - 11/31) and October 2022 (35.7% - 5/14). However, in some months the number of samples tested was very low and the positivity was as high as 100%, such as March/2020 (1/1) and January/2021 (1/1) (Figure 1).

Time trend analysis of prevalence, performed by regression analysis, showed that there was a drastic change in prevalence, with a decrease in January/2018 (MPC = –34 p<0.001) and an increase after March/2018 (MPC = 0.7 p<0.001; best fit model: one joinpoint).

All age groups showed NoV infections, but a considerable frequency was observed in children under two years of age, as it was inferred that children at this age were twice as likely to be affected by NoV gastroenteritis (33.3% - 98/294) (Table 1). Joinpoint regression analysis also showed a decreasing trend in prevalence according to patient age (APC = -16.2; p<0.0001) (Supplementary Figure S1).

**Table 1:** Frequency distribution of positive NoV cases by age group in samples from the Viral Gastroenteritis Surveillance Network dating from January 2018 to December 2022.

Age range (months/year)	Pos	Number of samples	Prevalence (%)	Odds ratio p-value
0 – 6m	14	60	23.33	O.R.=2.87 p<0.0001 NNH=6
6m – 12m	45	122	36.89	
12m – 24m	39	112	34.82	
24m – 5y	13	89	14.61	
5y – 10y	8	37	21.62	
10y – 19y	3	13	23.08	
>19y	7	70	10	
Total	129	503	-	

Pos = positive; m = months; y = years.

Considering the available information on norovirus epidemiological surveillance, 102 samples were selected for RT-PCR and sequencing. Of these, 52% (53/102) were amplified and sequenced (35 samples of the ORF1/ORF2 junction region and 18 of the capsid region).

**Figure 2:**
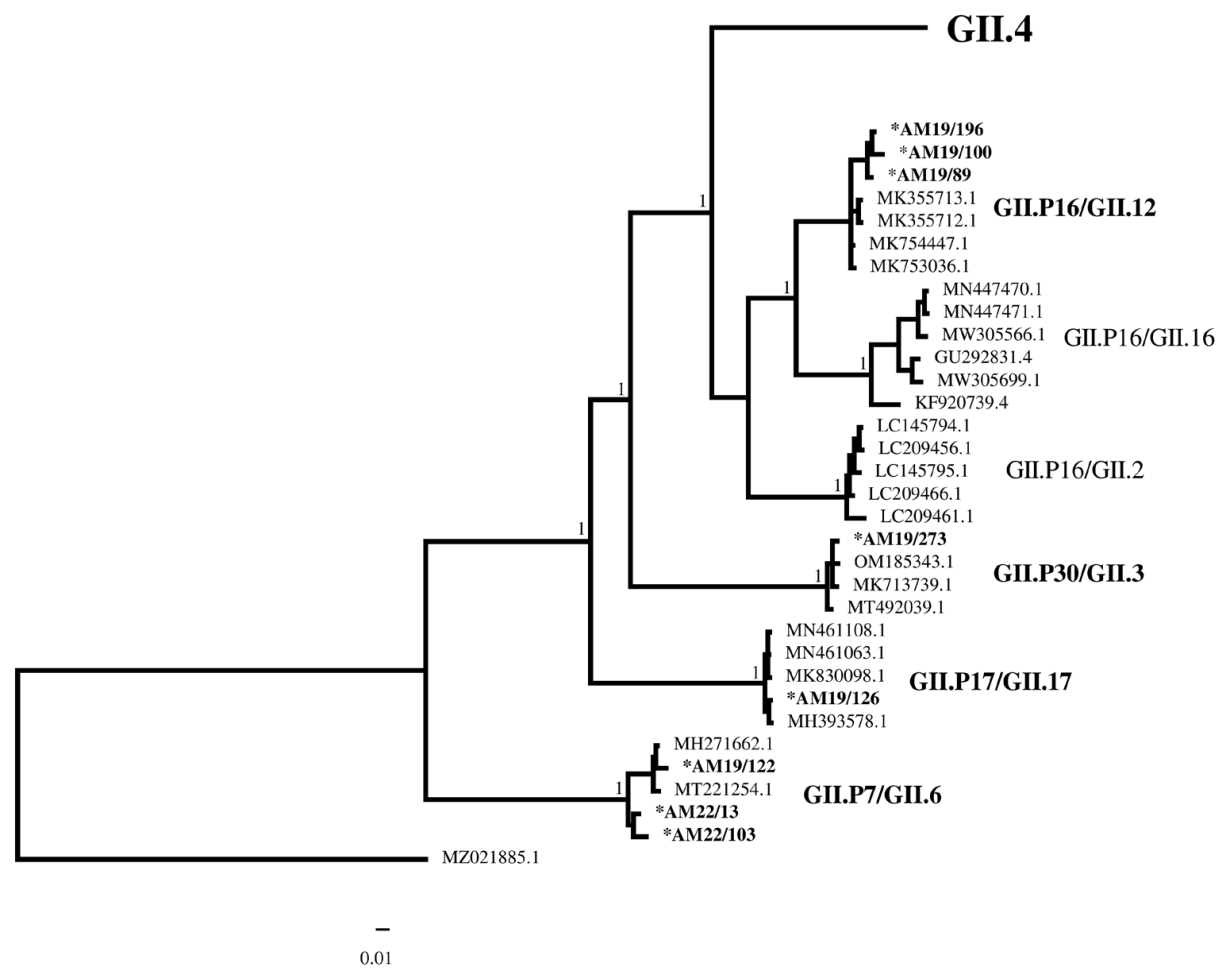
Phylogenetic trees of ORF1/ORF2 junction regions (544 bp) showing norovirus strains in samples from the Viral Gastroenteritis Surveillance Network, during the period 2018 to 2022. Evolutionary history was inferred by Bayesian analysis using the GTR substitution model in the Geneious program. NoV strains isolated in this study are shown in bold and marked with an asterisk.

In this study, most of the sequenced samples belonged to genogroup GII—98.1% (52/53) of the cases analyzed. Genogroup GI was identified in 1.9% (1/53) of the cases. The genotypes identified were: GII.P16/GII.4 (56.6% - 30/53); GII.P16/GII.12 (7.5% - 4/53); GII.P7/GII.6 (5.7% - 3/53); GII.P4/GII.4 (3.8% - 2/53); GII.P7/GII.7, GII.P30/GII.3, GII.P17/GII.17 and GII.P13/GI.3 (1.9% - 1/53). Among the samples genotyped by the ORF2 region only, GII.4 was detected in 17% (9/53) and GII.6 in 1.9% (1/53) (Figure 2). The RDP4 software (version 4.101, University of Cape Town, South Africa) was used to analyze the recombinant genotypes, using bootscan analysis (Model: Jukes and Cantor; window size 200; 100 bootstrap). Supplementary Figure S2 shows the results of the analysis.

**Figure 3:**
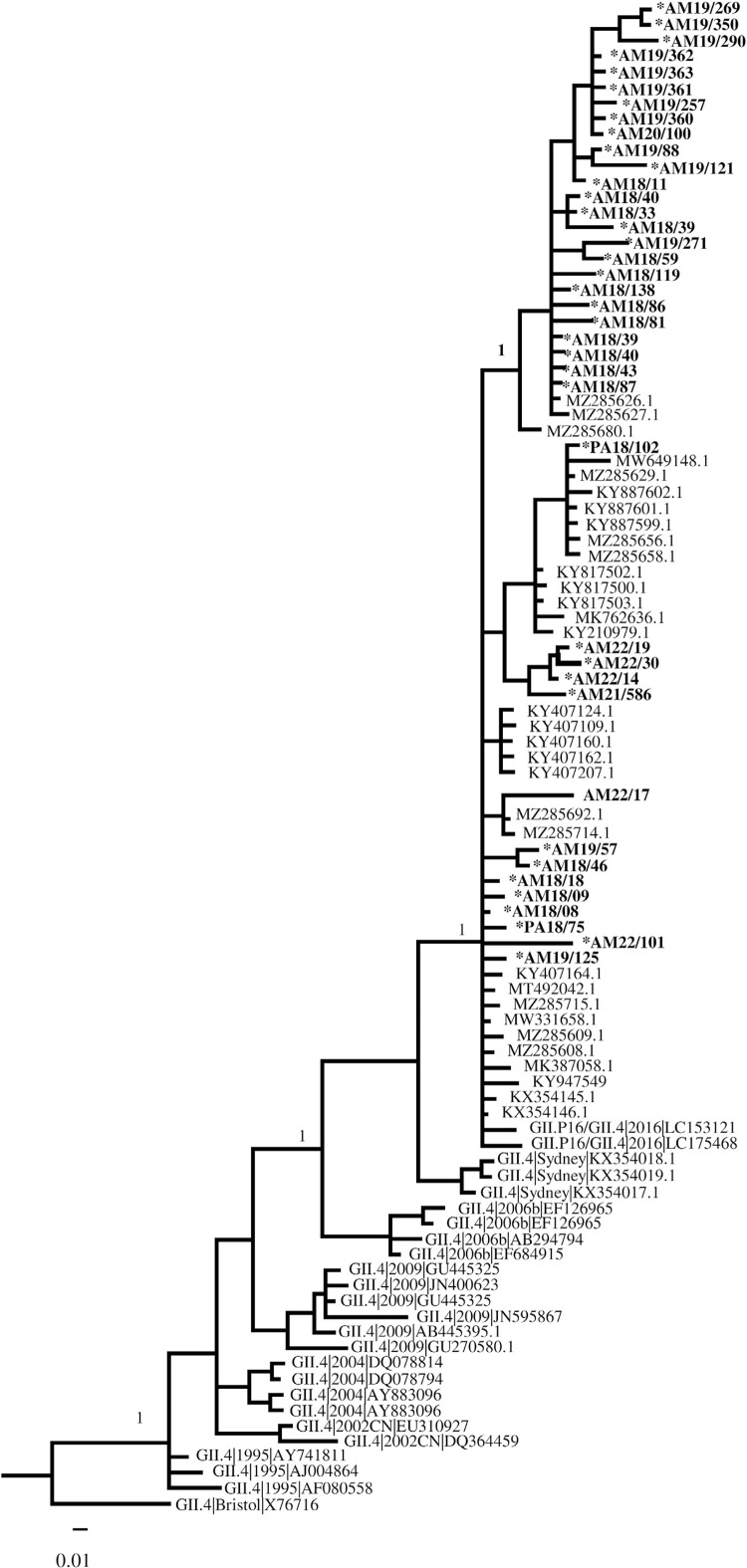
Phylogenetic trees of the capsid (390 bp) showing norovirus genotypes and molecular evolution of NoV-GII.4 strains in samples from the Viral Gastroenteritis Surveillance Network, during the period 2018 to 2022. Evolutionary history was inferred by Bayesian analysis using the GTR substitution model in the Geneious program. NoV strains isolated in this study are shown in bold and marked with an asterisk.

The phylogenetic tree of the capsid region shows the evolutionary aspects of the pandemic GII.4 variants circulating worldwide since 1995. The GII.P16/GII.4 strains are derived from the GII.4_Sydney strain (which emerged in 2012) and present as an ancestral variant of the Den Haag-2006b strain, which predominated during the years 2010-2012. BLASTn analysis showed that the GII.P16/GII.4 genotypes identified in this study share 95.6-99.7% identity with other strains of the same genotype circulating worldwide (Figure 3).

The RT-PCR assays validated using the protease region showed satisfactory specificity (100%) and sensitivity (100%). All samples of the GIIP16/GII.4 genotype amplified, while samples not belonging to the GII.4 genotype did not amplify. The GII.P16/GII.4 sequences obtained after sequencing the protease region (703 nucleotides) were used to construct a time-scaled phylogenetic tree inferred by Bayesian analysis using strict molecular clock models (best coalescent model after PS/SS sampling method: GMRF Bayesian skyride). The most recent common ancestor (TMRCA) of the GII.P16/GII.4 strains was estimated to have existed until 2012 (2011-2013 _ 95% HPD). The evolution rate of the pro region of norovirus GII.P16/GII.4 strains was estimated to be 2.87x10-3 nucleotide substitutions/site/year. Five clades were distinguished in this phylogeny, corresponding to the recombinant strains GII.P16/GII.2, GII.P16/GII.3, GII.P16/GII.13, GII.P16/GII.4 and GII.P16/GII.1. The GII.P16/GII.4 clade was closer to the GII.P16/GII.2 and GII.P16/GII.1 strains from China and Canada, respectively (Supplementary Figure S3). Supplementary Table S1 shows the dataset containing the genetic sequences from each branch of the molecular clock tree.

From the amino acid alignment of the Pro region sequences with other prototype strains circulating from 2008 to 2018, 13 nucleotide substitutions were observed, three of them (V→I, V→A, G→S) between the Brazilian strains detected in 2016 and the current strains (those from this study). The protease active sites (histidine 30, glutamate 54 and cysteine 139) were analyzed and no amino acid substitutions were detected (Supplementary Figure S4).

The sequences obtained in this study have been deposited in GenBank under the following accession numbers: OQ648027 - OQ648031; OQ648033 - OQ648054; OQ650231 - OQ650243.

## DISCUSSION

Noroviruses are recognized as one of the major causes of gastroenteritis outbreaks and sporadic cases globally. In the Amazon, several studies have demonstrated the incidence of NoV infection, with reported frequencies ranging from 12.3% to 38%^13,19-21^. The Amazon is characterized by urban, rural and riverside populations with limited access to treated water and proper sanitation, which ultimately contributes to the spread of waterborne diseases^21,22^.

NoV detection and molecular characterization were performed in the Amazon and the prevalence observed was 25.8%. This rate is higher than that reported in a study conducted in Rondonia (7.8%), which analyzed children under six years of age hospitalized for diarrhea^13^, and also higher than the rate reported in a study conducted in Rio Branco/Acre (12.3%), which assessed sporadic cases in patients with or without diarrhea^23^.

Other studies in the region have reported higher rates of positivity. For example, a study of patients hospitalized with acute gastroenteritis, conducted in Belem, Para State, from 2008 to 2010, found that the prevalence of gastroenteritis caused by NoV was 36.5%^24^. In addition, a study of sporadic cases in 2010 and 2011 found a prevalence of 35.2%^20^. In a study carried out in the southeastern region of Brazil, these viruses were found in 28.4% of 1,565 notifications of children with diarrhea admitted to sentinel units in the city of São Paulo from 2010 to 2016^12^.

NoV infections were found to be more prevalent in children under two years of age, who were potentially twice as likely as other age groups to be affected by NoV. Similar findings were reported in a survey of hospitalized children with symptoms of acute gastroenteritis, conducted in Qatar from 2016 to 2018. The survey found that NoV was present in 29.5% of cases, with the predominance of genotypes GII.4 and GII.3. The highest rate of infection was observed in children between the ages of 1 and 3 years old^25^. In a study of patients hospitalized for gastroenteritis in Bangladesh, it was found that 82% of NoV hospitalizations occurred in children under five years of age, indicating that young age and recent exposure to this virus are risk factors^8^. It is important to consider these findings when developing prevention and treatment strategies for this virus. This study confirms previous research suggesting an increased susceptibility to NoV infection in young children^12,13^.

NoV infections were detected in most months, with a decreasing trend in January 2018, followed by a resumption of the growth trend (but with lower frequency) in March of the same year. Consequently, from 2018 to 2019, NoV incidence decreased by 35.7%, which was accentuated the following year due to the SARS-CoV-2 pandemic, leading to a decrease in gastroenteric virus diagnoses.

Based on the data from this study, there was a decrease in the number of analyzed samples during the SARS-CoV-2 pandemic period in 2020. This was followed by an increease in cases of GA caused by NoV in 2021. The increased prevalence of this viral agent in 2021 may be attributed to the resumption of activities in crowded environments. This theory is supported by a study conducted by Lu *et al.* ^26^ that showed a decrease in outbreaks and sporadic NoV infections in Guangzhou, China, from January to August 2020. However, reported cases were higher after schools reopened in September 2020. Another study by Pham *et al.* ^27^ found that the COVID-19 pandemic was associated with a decrease in cases of NoV infection among infants and children in Japan from 2018 to 2021. This decrease may be due to pandemic control measures and hygiene practices.

The initial high prevalence in 2018 is thought to be a result of the increase in NoV cases associated with the emergence of the GII.P16/GII.4 variant in 2015. Significant changes in the prevalence of NoV infection have been observed from 2011 to this day. During this time, four pandemic NoV variants emerged, as identified in studies of the molecular epidemiology of NoV in the Viral Gastroenteritis Surveillance Network^20,28,29^.

The majority of NoV cases found in this study were caused by the GII.P16/GII.4 genotype, which was present in all years analyzed. Other common genotypes were: GII.P16/GII.12; GII.P7/GII.6; GII.P4/GII.4; and GII.P30/GII.3. The GII.P16/GII.4 pandemic strain emerged by recombination of the GII.P16 (polymerase) and GII.4_Sydney (capsid) variants. In Brazil, this genotype has undergone changes in the amino acid sequences of the nonstructural proteins p48, p22, and RdRp, as well as in the antigenic sites located in the P2 subdomain^10,29^.

The recombinant GII.P16/GII.4 strain has become the predominant genotype in certain regions of the world, replacing the GII.P31/GII.4 strain. GII.P31/GII.4 was the predominant genotype identified in previous studies conducted in the Amazon from 2010 to 2016^10^. In a recent survey conducted from 2019 to 2021 in rural areas of South Africa, GII.P31/GII.4 was found to cause 59% (19/32) of symptomatic infections in children with and without gastroenteritis^30^. Further studies indicate that GII.P16/GII.4 and GII.P31/GII.4 recombinants are the most prevalent genotypes globally^31^.

Results similar to those presented in this study were found in a survey conducted in Tehran, Iran. The GII.P16/GII.4 recombinant strain was found in 50% of samples from children hospitalized for acute gastroenteritis between 2021 and 2022^32^. In a NoV surveillance study conducted from January 2016 to March 2022 in children with gastroenteritis in Shanghai, NoV was detected in 301 (12.4%) of the 2,419 fecal samples analyzed. GII.P16/GII.4 predominated in 2021. Furthermore, 23 complete genomes of this recombinant strain were identified, indicating evolution into a new sublineage (SHGII.4-2020) with multiple mutations in non-structural proteins circulating in different regions of China^33^.

The second most prevalent recombinant genotype was GII.P16/GII.12. This strain was first identified in 2017 during investigations of outbreaks in the National Norovirus Outbreak Surveillance Network in the United States^34^. It was later found in Canada (May/2018), causing outbreaks and sporadic cases of gastroenteritis in children^35^. This strain was first detected in Brazil in October 2020 and associated with the consumption of NoV-contaminated ice cream^36^.

The GII.P7/GII.6 recombinant genotype was identified in three of the samples analyzed and was the third most frequent in this study. This genotype was found in six samples in Ningxia, China, from May to July 2020^37^. These findings suggest that new outbreaks may result from these genotypes. According to time-scale phylogenetic analyses, the RdRp region of the GII-P7 genotype and the VP1 region of the GII.6 genotype had evolutionary rates of 2.91 × 10^3^ and 2.61 × 10^3^, respectively. Based on a study that used sequences from GenBank, the evolutionary rates for globally collected GII.P6-GII.6 and GII.P7-GII.6 strains were 3.725 × 10^3^ and 3.482 × 10^3^, respectively^38^. The time-scale phylogeny indicated that the common ancestor of types P6 and P7 diverged approximately 50 years ago, while the common ancestor of type GII.6 diverged 110 years ago. These results suggest that genetic recombination played a crucial role in the evolution of this genotype.

The emergence of the unconventional recombinants GII.P30/GII.3 and GI.P13/GI.3 has been associated with outbreaks. The GI.P3/GI.3 genotype was detected in three samples from children under 5 years of age in Rio Branco, Acre, Brazil^13^.

In this study, the protease region was analyzed and the amplification perspectives were expanded using RT-PCR and evolutionary molecular analysis in neutral regions (without selective pressure). This region is fundamental for viral activity and is an important target for antiviral drugs^39^. The active sites of the enzyme operate by cleaving the polyprotein and encoding nonstructural proteins that assist in the formation of the replication complex^5^.

By using phylogenetic analyses and molecular clocks in this particular region, the evolutionary history could be reconstructed. Estimation methods were also used to identify the origin of the GII.P16/GII.4 strain. These strains evolved from GII.P16/GII.2 strains from China and GII.4_Sydney 2012 strains, indicating a common ancestor in 2012. In addition to these evolutionary parameters, four distinct clades were observed, representative of underlines corresponding to recombinant GII.P16 strains that emerged from 2009 to 2016^40^.

The GII.P16/GII.4 strains found in the region have an evolutionary rate of 2.87x10^3^ substitutions/site/year in the protease region. These strains originated from emerging strains that circulated in 2016. The ORF1 region was estimated to have a 2.01×10^3^substitutions/site/year evolutionary rate^29^.

The study has some limitations, such as the uneven distribution of sampling over time and the specific characteristics of the target population. Furthermore, the exclusion of samples with insufficient or exhausted fecal material must be considered. Nevertheless, this study provides a temporal trend of NoV prevalence from 2018 to 2022, with a detailed description of genotypic diversity and an evolutionary analysis of the protease gene. This neutral region plays a crucial role in viral activities and serves as an important target for antiviral drugs^39^. However, it is important to note that hypervariable regions within the viral genome, such as the P2 region, provide valuable information and must be analyzed accordingly^25^.

## CONCLUSION

This study revealed that NoV is one of the major causes of gastroenteritis in the Amazon. This underscores the importance of conducting epidemiological surveillance to prevent the spread of such infections. The study also revealed the widespread presence of recombinant and uncommon genotypes, improving the understanding of these viral genetic variations. This discovery could significantly assist in the development of antiviral medications and immunization.
