# Supplementary material for: Epidemiological and molecular surveillance of norovirus in the Brazilian Amazon: description of recombinant genotypes and improvement of evolutionary analysis
Source: Rev Inst Med Trop Sao Paulo. 2024 Apr 19;66:e22. doi: 10.1590/S1678-9946202466022 (PMC11027490; doi:10.1590/S1678-9946202466022)
Supplement: Supplementary file 1 [file 1678-9946-rimtsp-66-S1678-9946202466022-supl-mat.pdf]

REVISTA DO INSTITUTO DE MEDICINA TROPICAL DE SÃO PAULO

JOURNAL OF THE SÃO PAULO INSTITUTE OF TROPICAL MEDICINE

SUPPLEMENTARY MATERIAL

https://doi.org/10.48331/scielodata.LDLKGP

Epidemiological and molecular surveillance of norovirus in the Brazilian Amazon: description of recombinant genotypes and improvement of evolutionary analysis

Jonaia Novaes da Costa[1], Jones Anderson Monteiro Siqueira[1], Dielle Monteiro Teixeira[1], Patrícia dos Santos Lobo[1], Sylvia de Fátima dos Santos Guerra[1], Isadora Monteiro Souza[1], Bruna Trindade Moreira Cardoso[1], Luana Silva Soares Farias[1], Hugo Reis Resque[1], Yvone Benchimol Gabbay[1], Luciana Damascena da Silva[1]

[1]Instituto Evandro Chagas, Seção de Virologia, Ananindeua, Pará, Brazil

Correspondence to: Jonaia Novaes da Costa
Instituto Evandro Chagas, Seção de Virologia, Rodovia BR-316, KM 7 s/n, CEP 67030-000, Levilândia, Ananindeua, PA, Brazil

E-mail: jonaianovaes@gmail.com

Received: 3 October 2023

Accepted: 12 January 2024

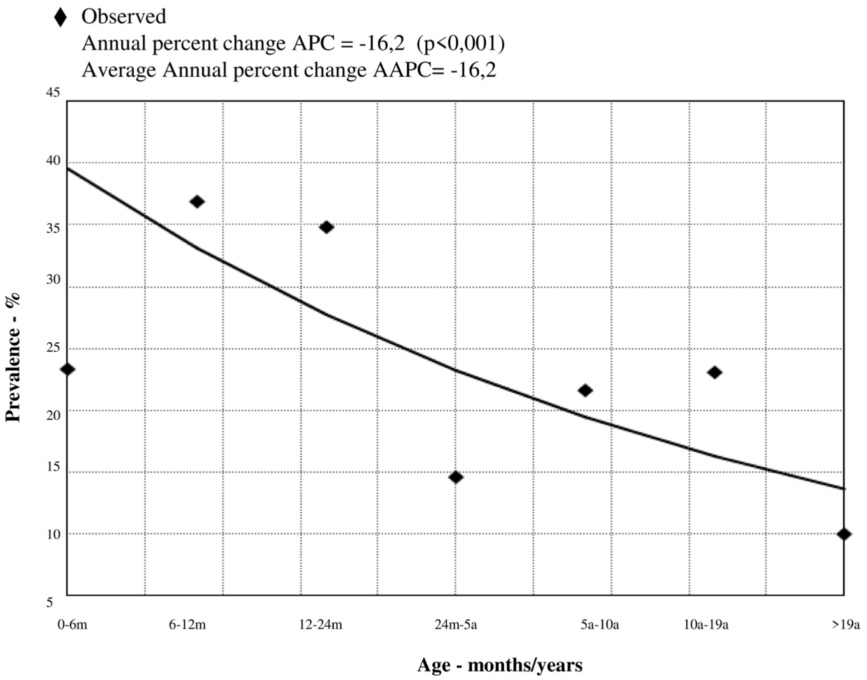

Supplementary Figure S1 - Distribution of the frequency of positive NoV cases by age group. Analysis performed on samples from the Viral Gastroenteritis Surveillance Network, during the period from January 2018 to December 2022. Parameters used in the analysis: minimum join point: 0 - max: 2; data-dependent model selection - Bayesian Information Criterion.

This is an open-access article distributed under the terms of the Creative Commons Attribution License.

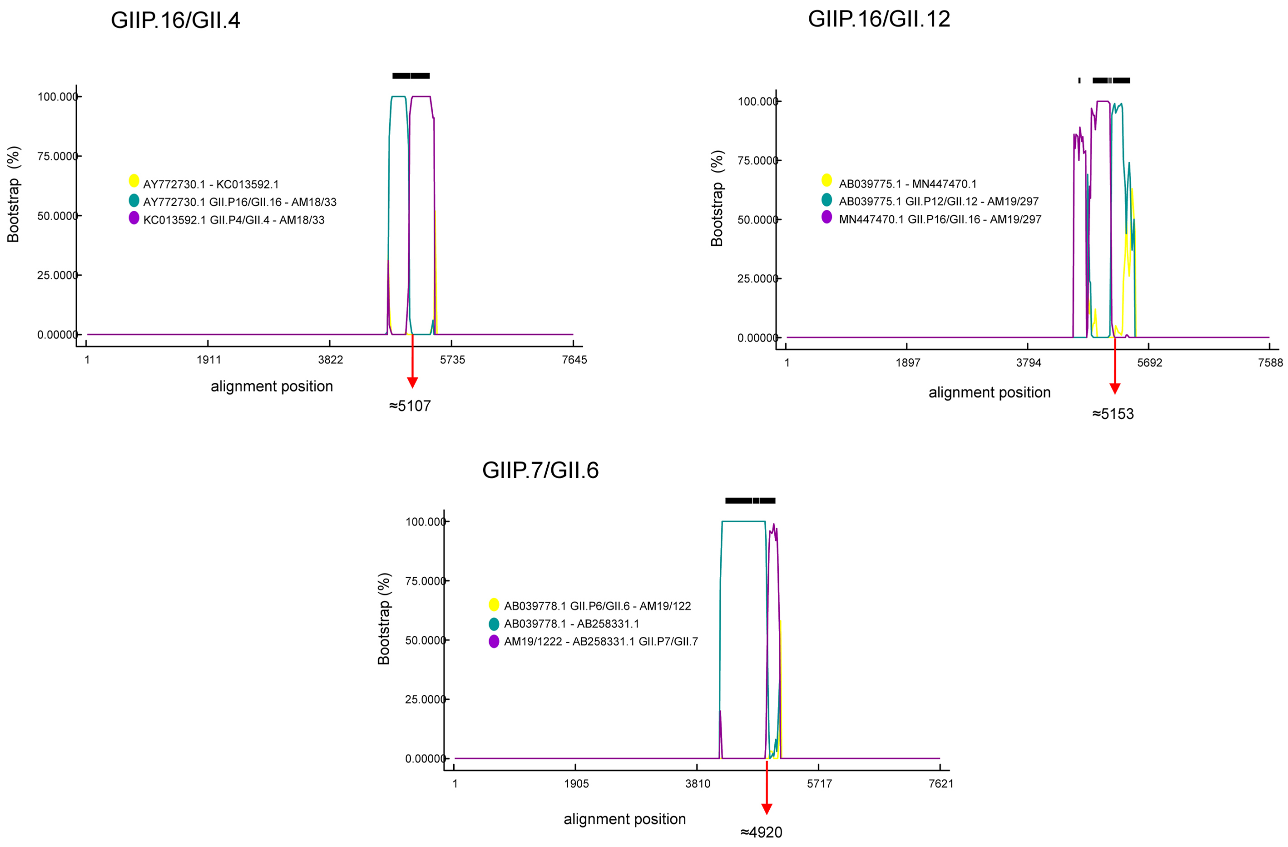

Supplementary Figure S2 - Graphs generated by the RDP4 program using alignments of the ORF1/2 overlapping region (512 bp) of the GII.P16/GII.4, GII.P16/GII.12 and GII.P7/GII.6 samples circulating in the Amazon from 2018 to 2022.

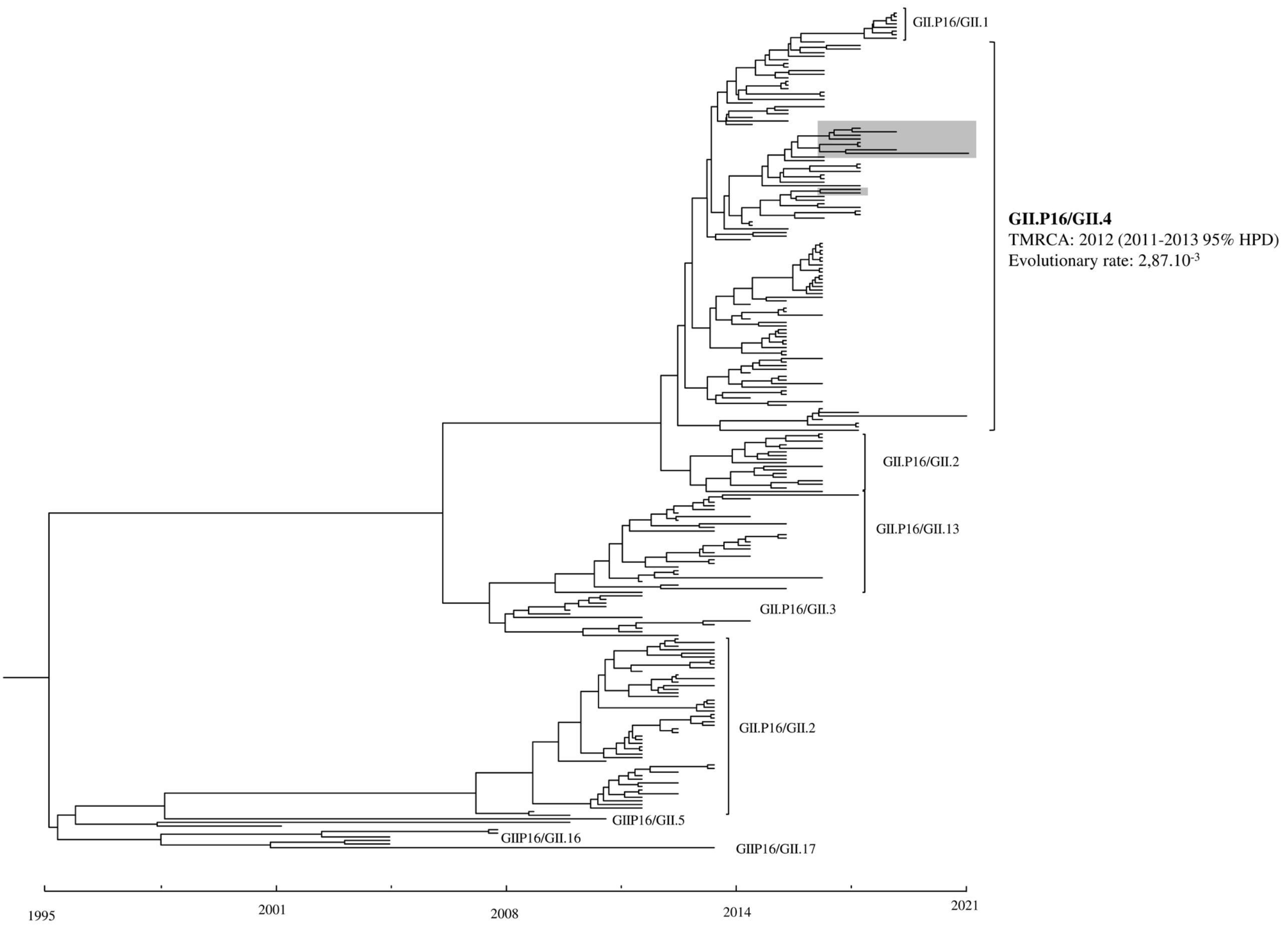

Supplementary Figure S3 - Time-scale phylogenetic tree of the protease region using a strict molecular clock, showing the dataset of 234 NoV-GII.P16 strains with samples from the Viral Gastroenteritis Surveillance Network collected during the period of 2018 to 2022. Evolutionary history was inferred by Bayesian analysis using MCMC in the Beast program. NoV strains isolated in this study are highlighted in gray.

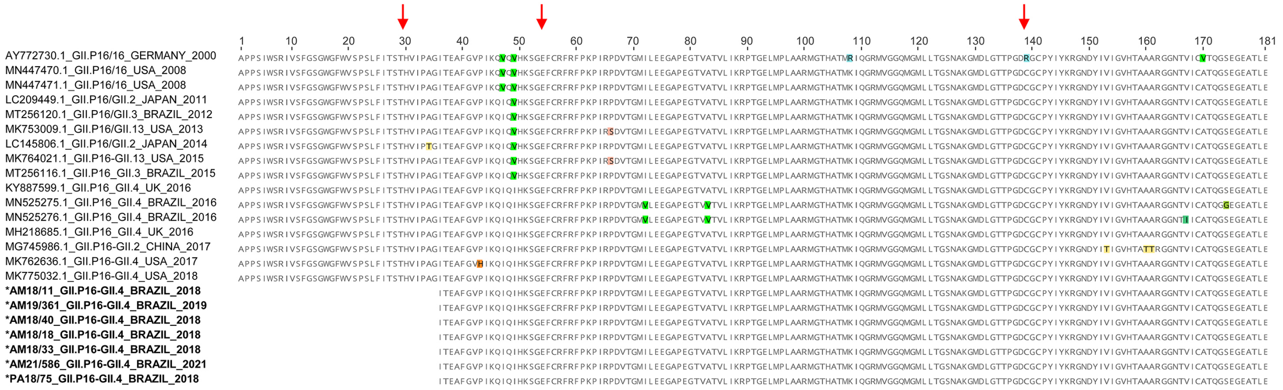

Supplementary Figure S4 - Alignment diagram describing the amino acids (146 AA) of the protease region (438 bp) of GII.P16/GII.4 norovirus strains circulating in the Amazon from 2018 to 2022. Colors indicate nucleotide substitution sites. Arrows indicate protease active sites.

Supplementary Table S1 - Database with information on the location and date of the genetic sequences used in elaboration the time-scale phylogeny of the protease region.

Sample name/ Nº Genbank access	Local	Year	Genotype
PA18/75	Brazil	2018	GII.P16/GII.4
AM18/40	Brazil	2018	GII.P16/GII.4
AM18/43	Brazil	2018	GII.P16/GII.4
AM21/586	Brazil	2021	GII.P16/GII.4
AM18/11	Brazil	2018	GII.P16/GII.4
AM19/361	Brazil	2019	GII.P16/GII.4
AM18/18	Brazil	2018	GII.P16/GII.4
AM18/33	Brazil	2018	GII.P16/GII.4
PA18/102	Brazil	2018	GII.P16/GII.4
**Nº Genbank access**	**Local**	**Year**	**Genotype**
AY772730.1	Germany	2000	GII.P16/GII.16
GU292831.4	Russia	2005	GII.P16/GII.16
HM596590.4	Russia	2010	GII.P16/GII.5
KC464505.1	Taiwan	2011	GII.P16/GII.2
KF920739.4	Russia	2012	GII.P16/GII.2
KJ196286.1	Japan	2002	GII.P16/GII.17
KM036380.1	Taiwan	2013	GII.P16/GII.13
KX907727.1	USA	2015	GII.P16/GII.4
KY210920.3	Russia	2016	GII.P16/GII.13
KY887597.1	UK	2016	GII.P16/GII.4
KY887598.1	UK	2016	GII.P16/GII.4
KY887599.1	UK	2016	GII.P16/GII.4
KY887600.1	UK	2016	GII.P16/GII.4
KY887601.1	UK	2016	GII.P16/GII.4
KY887602.1	UK	2015	GII.P16/GII.4
KY887604.1	UK	2015	GII.P16/GII.4
KY887605.1	UK	2015	GII.P16/GII.4
KY887606.1	UK	2016	GII.P16/GII.4
KY905335.1	Australia	2016	GII.P16/GII.4
KY947548.1	USA	2016	GII.P16/GII.13
KY947550.1	USA	2015	GII.P16/GII.4
LC122832.1	Japan	2012	GII.P16/GII.13
LC122839.1	Japan	2010	GII.P16/GII.2
LC122841.1	Japan	2014	GII.P16/GII.2
LC122843.1	Japan	2013	GII.P16/GII.2
LC122846.1	Japan	2014	GII.P16/GII.2
LC122849.1	Japan	2013	GII.P16/GII.2
LC122850.1	Japan	2012	GII.P16/GII.2
LC145786.1	Japan	2012	GII.P16/GII.2
LC145787.1	Japan	2012	GII.P16/GII.2
LC145788.1	Japan	2012	GII.P16/GII.2
LC145789.1	Japan	2012	GII.P16/GII.2
LC145790.1	Japan	2012	GII.P16/GII.2
LC145791.1	Japan	2012	GII.P16/GII.2
LC145792.1	Japan	2012	GII.P16/GII.2
LC145793.1	Japan	2012	GII.P16/GII.2
LC145794.1	Japan	2012	GII.P16/GII.2
LC145795.1	Japan	2012	GII.P16/GII.2
LC145796.1	Japan	2012	GII.P16/GII.2
LC145797.1	Japan	2012	GII.P16/GII.2
LC145798.1	Japan	2014	GII.P16/GII.2
LC145799.1	Japan	2014	GII.P16/GII.2
LC145800.1	Japan	2014	GII.P16/GII.2
LC145801.1	Japan	2014	GII.P16/GII.2
LC145802.1	Japan	2014	GII.P16/GII.2
LC145803.1	Japan	2014	GII.P16/GII.2
LC145804.1	Japan	2014	GII.P16/GII.2
LC145805.1	Japan	2014	GII.P16/GII.2
LC145806.1	Japan	2014	GII.P16/GII.2
LC145807.1	Japan	2014	GII.P16/GII.2
LC145808.1	Japan	2014	GII.P16/GII.2
LC175468.1	Japan	2016	GII.P16/GII.2
LC209431.1	Japan	2013	GII.P16/GII.2
LC209432.1	Japan	2012	GII.P16/GII.2
LC209433.1	Japan	2012	GII.P16/GII.2
LC209434.1	Japan	2014	GII.P16/GII.2
LC209441.1	Japan	2014	GII.P16/GII.2
LC209442.1	Japan	2013	GII.P16/GII.2
LC209443.1	Japan	2013	GII.P16/GII.2
LC209444.1	Japan	2013	GII.P16/GII.2
LC209445.1	Japan	2012	GII.P16/GII.2
LC209449.1	Japan	2011	GII.P16/GII.2
LC209450.1	Japan	2014	GII.P16/GII.2
LC209451.1	Japan	2011	GII.P16/GII.2
LC209454.1	Japan	2010	GII.P16/GII.2
LC209455.1	Japan	2013	GII.P16/GII.2
LC209456.1	Japan	2013	GII.P16/GII.2
LC209458.1	Japan	2014	GII.P16/GII.2
LC209460.1	Japan	2010	GII.P16/GII.2
LC209461.1	Japan	2009	GII.P16/GII.2
LC209466.1	Japan	2012	GII.P16/GII.2
LC209470.1	Japan	2014	GII.P16/GII.2
LC209475.1	Japan	2013	GII.P16/GII.2
LC209476.1	Japan	2013	GII.P16/GII.2
LC209477.1	Japan	2013	GII.P16/GII.2
LC209479.1	Japan	2011	GII.P16/GII.2
LC228948.1	Japan	2014	GII.P16/GII.2

Supplementary Table S1 - Database with information on the location and date of the genetic sequences used in elaboration the time-scale phylogeny of the protease region. (cont.)

LC597111.1	Indonesia	2015	GII.P16/GII.13
LC597113.1	Indonesia	2016	GII.P16/GII.13
LC597119.1	Indonesia	2015	GII.P16/GII.13
LC597122.1	Indonesia	2015	GII.P16/GII.13
LC597126.1	Indonesia	2016	GII.P16/GII.13
MG002630.1	Australia	2017	GII.P16/GII.4
MG745986.1	China	2017	GII.P16/GII.2
MG745995.1	China	2016	GII.P16/GII.2
MG746006.1	China	2017	GII.P16/GII.2
MG746009.1	China	2016	GII.P16/GII.2
MG746015.1	China	2016	GII.P16/GII.2
MG746017.1	China	2016	GII.P16/GII.2
MG746022.1	China	2017	GII.P16/GII.2
MG746023.1	China	2017	GII.P16/GII.2
MG746028.1	China	2016	GII.P16/GII.2
MG746029.1	China	2016	GII.P16/GII.2
MG746035.1	China	2016	GII.P16/GII.2
MG746036.1	China	2016	GII.P16/GII.2
MG746043.1	China	2017	GII.P16/GII.2
MG746044.1	China	2017	GII.P16/GII.2
MG746045.1	China	2016	GII.P16/GII.2
MG892953.3	Russia	2017	GII.P16/GII.3
MH218591.1	UK	2014	GII.P16/GII.17
MH218685.1	UK	2016	GII.P16/GII.4
MH260478.1	USA	2017	GII.P16/GII.4
MH260481.1	USA	2017	GII.P16/GII.4
MH260482.1	USA	2017	GII.P16/GII.4
MH260484.1	USA	2017	GII.P16/GII.4
MH260485.1	USA	2017	GII.P16/GII.4
MH260486.1	USA	2017	GII.P16/GII.4
MH260488.1	USA	2017	GII.P16/GII.4
MH260490.1	USA	2017	GII.P16/GII.4
MH260491.1	USA	2017	GII.P16/GII.4
MH260492.1	USA	2017	GII.P16/GII.4
MH260495.1	USA	2017	GII.P16/GII.4
MH260497.1	USA	2017	GII.P16/GII.4
MH260498.1	USA	2017	GII.P16/GII.4
MH260502.1	USA	2017	GII.P16/GII.4
MH260507.1	USA	2017	GII.P16/GII.4
MH260512.1	USA	2017	GII.P16/GII.4
MH279823.1	USA	2017	GII.P16/GII.4
MH279825.1	USA	2017	GII.P16/GII.4
MH702283.1	Bhutan	2014	GII.P16/GII.13
MK073885.1	USA	2016	GII.P16/GII.4
MK073888.1	USA	2016	GII.P16/GII.4
MK073890.1	USA	2016	GII.P16/GII.4
MK073891.1	USA	2016	GII.P16/GII.4
MK355712.1	Canada	2018	GII.P16/GII.4
MK355713.1	Canada	2018	GII.P16/GII.4
MK483908.2	Russia	2018	GII.P16/GII.4
MK483909.2	Russia	2018	GII.P16/GII.4
MK629457.1	USA	2016	GII.P16/GII.4
MK752933.1	USA	2018	GII.P16/GII.4
MK752934.1	USA	2018	GII.P16/GII.4
MK752936.1	USA	2017	GII.P16/GII.4
MK752937.1	USA	2017	GII.P16/GII.4
MK752942.1	USA	2016	GII.P16/GII.4
MK752943.1	USA	2016	GII.P16/GII.4
MK752946.1	USA	2015	GII.P16/GII.13
MK752947.1	USA	2014	GII.P16/GII.13
MK752949.1	Japan	2013	GII.P16/GII.2
MK753008.1	USA	2013	GII.P16/GII.13
MK753009.1	USA	2013	GII.P16/GII.13
MK753010.1	USA	2014	GII.P16/GII.13
MK753017.1	USA	2017	GII.P16/GII.2
MK753018.1	USA	2017	GII.P16/GII.4
MK753020.1	USA	2014	GII.P16/GII.13
MK753029.1	USA	2016	GII.P16/GII.4
MK753030.1	USA	2015	GII.P16/GII.4
MK753032.1	USA	2016	GII.P16/GII.4
MK753033.1	USA	2016	GII.P16/GII.1
MK753034.1	USA	2017	GII.P16/GII.4
MK753036.1	USA	2017	GII.P16/GII.4
MK754442.1	USA	2015	GII.P16/GII.4
MK754443.1	USA	2014	GII.P16/GII.13
MK754444.1	USA	2017	GII.P16/GII.4
MK754446.1	USA	2018	GII.P16/GII.4
MK754447.1	USA	2018	GII.P16/GII.4
MK756033.1	USA	2016	GII.P16/GII.4
MK762559.1	Puerto Rico	2014	GII.P16/GII.13
MK762560.1	Puerto Rico	2014	GII.P16/GII.13
MK762562.1	USA	2016	GII.P16/GII.4
MK762563.1	USA	2016	GII.P16/GII.4
MK762564.1	USA	2016	GII.P16/GII.4
MK762566.1	USA	2016	GII.P16/GII.4
MK762569.1	USA	2017	GII.P16/GII.4
MK762570.1	USA	2017	GII.P16/GII.4
MK762621.1	USA	2017	GII.P16/GII.4
MK762627.1	USA	2017	GII.P16/GII.4
MK762629.1	USA	2017	GII.P16/GII.4

Supplementary Table S1 - Database with information on the location and date of the genetic sequences used in elaboration the time-scale phylogeny of the protease region. (cont.)

MK762635.1	USA	2017	GII.P16/GII.4
MK762636.1	USA	2017	GII.P16/GII.4
MK762637.1	USA	2015	GII.P16/GII.4
MK762745.1	USA	2018	GII.P16/GII.13
MK764013.1	USA	2016	GII.P16/GII.4
MK764014.1	USA	2015	GII.P16/GII.13
MK764015.1	USA	2017	GII.P16/GII.4
MK764018.1	USA	2016	GII.P16/GII.4
MK764021.1	Brazil	2018	GII.P16/GII.4
MK764021.1	USA	2017	GII.P16/GII.2
MK773584.1	USA	2016	GII.P16/GII.4
MK773585.1	USA	2016	GII.P16/GII.4
MK775030.1	USA	2017	GII.P16/GII.4
MK775031.1	USA	2017	GII.P16/GII.4
MK775032.1	USA	2018	GII.P16/GII.4
MN447470.1	USA	2008	GII.P16/GII.16
MN447471.1	USA	2008	GII.P16/GII.16
MN525275.1	Brazil	2016	GII.P16/GII.4
MN525276.1	Brazil	2016	GII.P16/GII.4
MN897756.1	USA	2018	GII.P16/GII.4
MN996297.1	China	2018	GII.P16/GII.4
MN996299.1	China	2018	GII.P16/GII.4
MT031761.1	USA	2017	GII.P16/GII.4
MT238666.1	USA	2017	GII.P16/GII.4
MT256116.1	Brazil	2015	GII.P16/GII.3
MT256120.1	Brazil	2012	GII.P16/GII.3
MT316113.1	Brazil	2012	GII.P16/GII.3
MT316122.1	Brazil	2014	GII.P16/GII.3
MT344179.1	USA	2015	GII.P16/GII.4
MT526278.1	USA	2014	GII.P16/GII.13
MT720841.1	USA	2015	GII.P16/GII.4
MW305566.1	Paraguay	2005	GII.P16/GII.16
MW305647.1	Argentina	2017	GII.P16/GII.4
MW305678.1	Japan	2013	GII.P16/GII.13
MW305699.1	Thailand	2005	GII.P16/GII.16
MW305714.1	Thailand	2012	GII.P16/GII.13
MW305723.1	Thailand	2018	GII.P16/GII.4
MW661254.1	Canada	2016	GII.P16/GII.4
MW661263.1	Canada	2019	GII.P16/GII.1
MW661264.1	Canada	2019	GII.P16/GII.4
MW661266.1	Canada	2019	GII.P16/GII.1
MW661267.1	Canada	2019	GII.P16/GII.1
MW661268.1	Canada	2019	GII.P16/GII.1
MW661269.1	Canada	2019	GII.P16/GII.1
MW661270.1	Canada	2019	GII.P16/GII.1
MW661271.1	Canada	2019	GII.P16/GII.1
MW661272.1	Canada	2019	GII.P16/GII.1
MZ268407.1	USA	2013	GII.P16/GII.13
MZ268408.1	USA	2013	GII.P16/GII.13
MZ478136.1	Mexico	2016	GII.P16/GII.4
MZ478139.1	Mexico	2016	GII.P16/GII.4
MZ478140.1	Mexico	2017	GII.P16/GII.4
MZ478141.1	Mexico	2017	GII.P16/GII.4
MZ958411.1	Russia	2017	GII.P16/GII.4
NC_039477.1	UK	2016	GII.P16/GII.4
OL898512.1	USA	2015	GII.P16/GII.4
OL898514.1	USA	2016	GII.P16/GII.4
OL943801.1	Guatemala	2013	GII.P16/GII.3
OP609646.1	Bangladesh	2011	GII.P16/GII.16
OP690427.1	USA	2021	GII.P16/GII.4
**Sample name**	**Local**	**Year**	**Genotype**
AM18/08	Brazil	2018	GII.P16/GII.4
AM18/09	Brazil	2018	GII.P16/GII.4
AM18/11	Brazil	2018	GII.P16/GII.4
AM18/18	Brazil	2018	GII.P16/GII.4
AM18/33	Brazil	2018	GII.P16/GII.4
AM18/39	Brazil	2018	GII.P16/GII.4
AM18/40	Brazil	2018	GII.P16/GII.4
AM18/86	Brazil	2018	GII.P16/GII.4
AM18/87	Brazil	2018	GII.P16/GII.4
AM18/119	Brazil	2018	GII.P16/GII.4
AM18/138	Brazil	2018	GII.P16/GII.4
PA18/75	Brazil	2018	GII.P16/GII.4
PA18/102	Brazil	2018	GII.P16/GII.4
AM19/89	Brazil	2019	GII.P16/GII.12
AM19/100	Brazil	2019	GII.P16/GII.12
AM19/122	Brazil	2019	GII.P7/GII.6
AM19/126	Brazil	2019	GII.17
AM19/196	Brazil	2019	GII.P16/GII.12
AM19/257	Brazil	2019	GII.P16/GII.4
AM19/266	Brazil	2019	GII.4
AM19/273	Brazil	2019	GII.P30/GII.3
AM19/360	Brazil	2019	GII.P16/GII.4
AM19/361	Brazil	2019	GII.P16/GII.4
AM19/362	Brazil	2019	GII.P16/GII.4
AM19/363	Brazil	2019	GII.P16/GII.4
AM21/586	Brazil	2021	GII.P16/GII.4
AM22/13	Brazil	2022	GII.P7/GII.6
AM22/17	Brazil	2022	GII.P16/GII.4
AM22/19	Brazil	2022	GII.P16/GII.4

Supplementary Table S1 - Database with information on the location and date of the genetic sequences used in elaboration the time-scale phylogeny of the protease region. (cont.)

AM22/30	Brazil	2022	GII.P16/GII.4
AM22/101	Brazil	2022	GII.P16/GII.4
AM22/103	Brazil	2022	GII.P7/GII.6
**Nº Genbank access**	**Local**	**Year**	**Genotype**
MT725762.1	USA	2017	
OL456442.1	Belarus	2021	GII.P7/GII.6
OL456443.1	Belarus	2021	GII.P7/GII.6
MZ269085.1	Belarus	2020	GII.P7/GII.6
MZ269086.1	Belarus	2020	GII.P7/GII.6
MH271662.1	Brazil	2020	GII.P7/GII.6
MT221254.1	Brazil	2014	GII.P7/GII.6
MZ044974.1	Brazil	2015	GII.P4-NewOrleans_2009/GII.4-Sydney2012
MZ044975.1	Brazil	2018	GII.P4-NewOrleans_2009/GII.4-Sydney2012
MT362913.1	USA	2017	GII.P4-NewOrleans_2009/GII.4-Sydney2012
MW009070.1	USA	2017	GII.P4-NewOrleans_2009/GII.4-Sydney2012
MT362912.1	USA	2017	GII.P4-NewOrleans_2009/GII.4-Sydney2012
MZ285680.1	Brazil	2018	GII.P16/GII.4
MW331658.1	India	2015	GII.P16/GII.4
MT501815.1	Spain	2019	GII.P16/GII.4
GU292831.4	Russia	2005	GII.P16/GII.16
MW305699.1	Thailand	2005	GII.P16/GII.16
MN447470.1	USA	2018	GII.P16/GII.16
MN447471.1	USA	2018	GII.P16/GII.16
MW305566.1	Paraguay	2005	GII.P16/GII.16
KF920739.4	Russia	2012	GII.P16/GII.16
MK754447.1	USA	2018	GII.P16/GII.12
MK753036.1	USA	2017	GII.P16/GII.12
MK754445.1	USA	2017	GII.P16/GII.12
MK616559.1	USA	2018	GII.P16/GII.12
MK355713.1	Canada	2018	GII.P16/GII.12
MK355712.1	Canada	2018	GII.P16/GII.12
LC145794.1	Japan	2012	GII.P16/GII.2
LC209456.1	Japan	2013	GII.P16/GII.2
LC145795.1	Japan	2012	GII.P16/GII.2
LC209466.1	Japan	2012	GII.P16/GII.2
LC209458.1	Japan	2014	GII.P16/GII.2
LC209461.1	Japan	2009	GII.P16/GII.2
LC209460.1	Japan	2010	GII.P16/GII.2
MK713739.1	Hong Kong	2019	GII.P30/GII.3
OM185345.1	Spain	2019	GII.P30/GII.3
OM185346.1	Spain	2019	GII.P30/GII.3
MT492039.1	Spain	2019	GII.P30/GII.3
OM185343.1	Spain	2018	GII.P30/GII.3
MH842217.1	China	2017	GII.P17/GII.17
MT238665.1	USA	2015	GII.P17/GII.17
MN461108.1	South Korea	2016	GII.P17/GII.17
LC369255.1	Japan	2015	GII.P17/GII.17
MK830097.1	Russia	2018	GII.P17/GII.17
MZ021885.1	South Korea	2018	GI.P1/GI.1
